# Raspberry, not a car: context predictability and a phonological advantage in early and late learners’ processing of speech in noise

**DOI:** 10.3389/fpsyg.2014.01449

**Published:** 2014-12-19

**Authors:** Kira Gor

**Affiliations:** Graduate Program in Second Language Acquisition, School of Languages, Literatures, and Cultures, University of Maryland, College ParkMD, USA

**Keywords:** heritage language speakers, speech in noise, early and late learners, second language acquisition, language proficiency, non-native speech recognition, context predictability, phonological sensitivity

## Abstract

Second language learners perform worse than native speakers under adverse listening conditions, such as speech in noise (SPIN). No data are available on heritage language speakers’ (early naturalistic interrupted learners’) ability to perceive SPIN. The current study fills this gap and investigates the perception of Russian speech in multi-talker babble noise by the matched groups of high- and low-proficiency heritage speakers (HSs) and late second language learners of Russian who were native speakers of English. The study includes a control group of Russian native speakers. It manipulates the noise level (high and low), and context cloze probability (high and low). The results of the SPIN task are compared to the tasks testing the control of phonology, AXB discrimination and picture-word discrimination, and lexical knowledge, a word translation task, in the same participants. The increased phonological sensitivity of HSs interacted with their ability to rely on top–down processing in sentence integration, use contextual cues, and build expectancies in the high-noise/high-context condition in a bootstrapping fashion. HSs outperformed oral proficiency-matched late second language learners on SPIN task and two tests of phonological sensitivity. The outcomes of the SPIN experiment support both the early naturalistic advantage and the role of proficiency in HSs. HSs’ ability to take advantage of the high-predictability context in the high-noise condition was mitigated by their level of proficiency. Only high-proficiency HSs, but not any other non-native group, took advantage of the high-predictability context that became available with better phonological processing skills in high-noise. The study thus confirms high-proficiency (but not low-proficiency) HSs’ nativelike ability to combine bottom–up and top–down cues in processing SPIN.

## INTRODUCTION

### WHO ARE HERITAGE SPEAKERS?

More people in the world are raised bilingual or multilingual than monolingual (Bhatia and Ritchie, 2013, XXI). Among the millions of bilingual speakers across the world, there is a group that have been called heritage speakers (HSs). HSs are early interrupted learners, who acquire their first language naturalistically as infants at home from their caregivers, but who switch to the language spoken in the community in their childhood (Valdés, 2005; Polinsky, 2008). As a result, second language (L2) becomes the dominant language of HSs, and their first (L1), heritage, language is reduced to non-native levels of proficiency due to incomplete acquisition and/or attrition (Montrul, 2008; Bylund, 2009; Bylund et al., 2010; Schmid, 2010; Polinsky, 2011). The heritage language may also be influenced by L2, the dominant language (Cook, 2003; Polinsky, 2014). HSs rely predominantly on auditory input, and often do not go through formal schooling in their first language. Due to this auditory bias, they typically prefer the listening and speaking modalities, have poor reading and writing skills, and are sometimes illiterate. HSs, early starters with non-native proficiency in their first language, have recently attracted the attention of researchers. And indeed, understanding the role of early start (from birth) in shaping the linguistic profile and the underlying processing mechanisms of HSs as opposed to late L2 starters makes it possible to address the critical period hypothesis (Abrahamsson and Hyltenstam, 2009; Bylund et al., 2012; DeKeyser, 2013). At the same time, HSs are compared to native speakers since both populations acquire language naturalistically from birth. This allows researchers to identify native and non-native aspects of heritage language (Montrul, 2012), and to establish the role of incomplete acquisition as opposed to attrition (Bylund et al., 2010).

Late L2 learners, unlike heritage language speakers, start learning their second language as adults, after puberty. The type of L2 exposure, naturalistic or formal classroom, depends on biographic trajectories of individual L2 learners, and on global migration patterns for larger populations of learners. Demographic trends, including the patterns of migration, often determine which populations of L2 learners will study L2 in a foreign language classroom, and which will actually move to the country where L2 is spoken. Formal late L2 learners, and university students in particular, often rely heavily on visual input (Psaltou-Joyceya and Kantaridoub, 2011). While there exists a range of methodologies for teaching a foreign language to late learners in a classroom setting outside the target language community, university-level academic programs in the U.S. typically introduce reading in Russian from the outset (Gor, 2000). A perusal of the major Russian language textbooks for beginners currently used in American universities shows that they rely on reading from day 1 (Lubensky et al., 2002; Lekic et al., 2008; Robin et al., 2014). In this study, native speakers of American English and late L2 learners of Russian were all predominantly shaped by in-class experience, which could be complemented by an immersion. No late L2 learner in the sample was a naturalistic learner. Conversely, HSs acquire their heritage language from birth in a uniquely auditory modality. Research on HSs in comparison with adult native speakers and late L2 learners makes it possible to gage the role of early naturalistic exposure in shaping the mechanisms underlying auditory speech processing. The uniqueness of HSs lies in the fact that they have received early naturalistic input in the same way as native speakers, yet have reduced, non-native proficiency in their L1, and thus can be compared to late L2 learners at the same proficiency level to single out the influence of early naturalistic exposure and input.

To summarize, heritage language is a native language acquired naturalistically from birth from caregivers that does not reach native proficiency levels due to a switch to another language spoken in the community, which becomes the dominant language. Heritage languages are often spoken languages due to the reduced amount of schooling that heritage language speakers receive. While there is a growing number of studies addressing the domains of heritage language phonology (Oh et al., 2003; Chang et al., 2011; Lukyanchenko and Gor, 2011), morphology (Gor et al., 2009; Gor and Cook, 2010), morphosyntax (Montrul et al., 2008, 2013, 2014; Montrul, 2009, 2011), and syntax (Keating et al., 2011; Lee-Ellis, 2011; Polinsky, 2011), there have been no studies, to the best of our knowledge, exploring the robustness of heritage auditory sentence processing, and in particular, HSs’ ability to rely on context predictability in adverse conditions, such as speech in noise (SPIN).

### SPEECH IN NOISE AND TOP–DOWN AND BOTTOM–UP PROCESSING

Given that SPIN, as one of the adverse conditions, has been used to study the properties of the human speech recognizer (Mattys et al., 2012), it can become a powerful diagnostic tool for the robustness of non-native speech perception. Moreover, recent renewed interest in speech processing in adverse conditions, including different kinds of noise, stems from the understanding that (1) adverse conditions are ecologically more valid than unrealistic idealized listening conditions, e.g., clear speech (see Mattys et al., 2012), and (2) by manipulating the properties of noise and the listening materials, one gains insights into the complex interaction of top–down and bottom–up processing in different groups of listeners. Was it raspberry or car (‘malina’ or ‘mashina,’ correspondingly, in Russian) that was mentioned in the sentence? In noisy conditions, these two feminine nouns can be confused easily. However, the context in which they were heard usually disambiguates the word in question. The high cloze probability context, if recovered from noise, will disambiguate *car* and *raspberry* in Russian sentences 1a and 1b.

(1a) Okolo doma stojala staraja mashina.

   Near house stood old car.NOM.SG.

   ‘An old car stood near the house.’

(1b) V sadu rosla spelaja malina.

   In garden grew ripe raspberry.NOM. SG.

   ‘Ripe raspberries grew in the garden.’

Critically, the whole sentence is masked by noise, and not just the last word, and the listener therefore needs to recover sentence cues from the acoustically degraded signal. This means that the mechanisms of prediction and sentence integration need to rely on acoustic cues that are less than robust, starting from the beginning of the sentence and building up expectations by the last word. Note that Russian allows scrambling, but crucially, the word order with the sentence-final noun-subject is canonical for this particular sentence structure, with the adverbial phrase fronted. Context predictability was manipulated in the original SPIN test developed for native speakers of English (Kalikow et al., 1977) and later adapted for Spanish (Cervera and González-Alvarez, 2011). The role of prediction and its interaction with heritage and late L2 learner profiles and high/low-proficiency levels is the main focus of the present study.

### NOISE TYPES AND INFORMATIONAL AND ENERGETIC MASKING

Before we address non-native processing of SPIN, let us revisit the understanding of the impact of different types of environmental degradation, including noise, on speech processing in native speakers. This will assist us in situating the present study and later in interpreting the findings with regard to the type of the noise that it used. There are two types of environmental degradation that are used in psycholinguistic experiments: energetic masking and informational masking (Van Engen and Bradlow, 2007; see Mattys et al., 2012 for a review). Energetic masking is created by the use of white noise or filtering and requires signal separation and lower-level acoustic encoding and activation of lexical-semantic information. Conversely, informational masking such as babble noise or speech compression interferes with higher-order selection and integration (Aydelott and Bates, 2004). The study by Aydelott and Bates (2004) used two types of distortion, low-pass filtering and 50% speech compression, and three types of priming sentence context, congruent, incongruent, and neutral. The format of the experiment was a lexical decision task with priming, where the priming context was manipulated, and the target final word (or non-word) was presented without distortions. The study recorded reduced facilitation in congruent low-pass filtered sentences, and reduced inhibition in incongruent compressed sentences compared to the neutral context. It concluded that energetic masking induced by low-pass filtering interfered with early low-level acoustic encoding and the activation of lexical entries, while sentence compression affected central language processing and sentence integration. While, there are no data at present on the impact of different adverse conditions on HSs’ speech recognition, it is reasonable to assume that the involvement of different levels of speech processing depending on the type of distortion will be same as for native speakers.

The present study used a multi-talker babble noise, which sounds like the noise of many people talking at the same time in the background. This type of noise is ecologically valid given its pervasive presence in everyday life. Note that listening to speech in adverse conditions is considered to be part of a listener’s daily auditory experience rather than an extraordinary situation, and consequently, Mattys et al. (2012, p. 963) maintain that speech recognition in adverse conditions is synonymous with speech recognition *per se*. Thus, SPIN tests the robustness of non-native listeners’ speech recognition under ecologically valid conditions.

Multi-talker babble noise combines both energetic and informational masking and thereby has a double effect on speech intelligibility. The superposition of several speech recordings on the target sentence produced a white noise component that is associated with energetic masking (Mattys et al., 2012). Energetic masking, as well as low-pass filtering, primarily affects the acoustic-phonetic properties of speech, and decreases its intelligibility by interfering with low-level processing. The more talkers, the more energetic masking takes place. At the same time, once the informational masking effect is partialled out, babble noise also produces informational masking that has different implications for speech intelligibility. Informational masking has higher-level consequences, as it leads to attentional capture, semantic interference, and eventually, increases the cognitive load. In the present study, the multi-talker babble had a high component of steady noise, but it also had an informational masking component, with a more limited competition between the informational streams than in a two-talker babble.

### SPEECH IN NOISE IN NON-NATIVE PERCEPTION

There exists a large body of evidence that L2 speakers’ perception of L2 speech in noisy conditions deteriorates to a greater extent than does the perception of native speakers (Kalikow et al., 1977; Mayo et al., 1997; Munro, 1998; van Wijngaarden et al., 2002). This effect has possible explanations involving redundancy reduction or fuzziness in L2 perception at different levels, from phonetic (e.g., uncertainty about phonetic contrasts) to semantic. Apparently L2 speakers do not make efficient use of the probabilities that context provides. “The levels of noise at which the speech was intelligible were significantly higher and the benefit from context was significantly greater for monolinguals … than for late bilinguals” (Mayo et al., 1997, p. 686).

While there is numerous evidence that non-native speech perception is affected by noisy conditions to a greater extent than native perception, there is no agreement regarding the relative role of several factors implicated in L2 learners’ perceptual problems when processing SPIN. Reduced speech discriminability in SPIN has been demonstrated in L2 listeners for non-word syllables (Cutler et al., 2004, 2008; Rogers et al., 2006; Broersma and Scharenborg, 2010), isolated words presented in lists (Rogers et al., 2006), words embedded in a sentence (Mayo et al., 1997; Bradlow and Alexander, 2007; Oliver et al., 2012), and whole sentences (Meador et al., 2000; Bradlow and Bent, 2002; Pinet et al., 2011). Studies focusing on the role of different aspects of non-native speech processing affected by noise fall mainly into three categories. The first category focuses on sublexical processing of isolated phonemes, e.g., individual phonemic confusions for English intervocalic consonants (Garcia Lecumberri and Cooke, 2006; Cutler et al., 2008; Broersma and Scharenborg, 2010). The second category is concerned with the phonological/lexical interface and phonemic confusions associated with word recognition (Oyama, 1982; Meador et al., 2000; Cooke et al., 2008). And finally, the third explores the reliance on sentence context and the use of cloze probabilities (van Wijngaarden et al., 2002; Bradlow and Alexander, 2007). The priming role of the context presented in noise in native and non-native populations has been explored for word priming (Golestani et al., 2009, 2013; Hervais-Adelman et al., 2014), and sentence priming (Aydelott and Bates, 2004). Crucially, two studies exploring the behavioral and neural bases of semantic context use in word and sentence priming, showed a consistent semantic context advantage for native speakers, but not second language learners (Golestani et al., 2009, 2013; Hervais-Adelman et al., 2014).

Studies explore the use of sentence context and cloze probabilities in various ways. The SPIN test (Kalikow et al., 1977) compared recognition of the sentence-final word, with the preceding context either making the word highly probable or impossible to predict. Thus, if at least part of the sentence can be auditorily recovered from noise in ‘The mouse was caught in the trap,’ the listener is unlikely to hear ‘tram’ instead of ‘trap.’ At the same time, when the context does not support the choice of one word over the other, confusion is more likely to occur. In: ‘They hope he heard about the rent,’ the low cloze probability does not support either the actual or the alternative word, for example, ‘tent.’ A more radical approach to cloze probabilities was adopted by Meador et al. (2000) who created sentences with low transitional probabilities between each word in the sentence and the following one, as in: ‘The blonde dentist ate the heavy bread.’ There, participant’s task was to repeat the sentence verbatim, and the accuracy score referred to the number of words that were correctly recovered from the sentence. The present study uses the approach of Kalikow et al. (1977), with two types of sentences differing by the probability of the last word only, which makes it possible to control for the properties of sentence-final words recognized in noise.

A study by Meador et al. (2000) directly addressed the relative role of non-native phonology in non-native word recognition in sentences. The study hypothesized that the native Italian participants’ accuracy in perceiving English vowels and consonants would be related to their recognition of English words in sentences with low transitional probabilities between words, as in the example above. To verify this hypothesis, the authors regressed the segmental perception scores obtained for the native Italian participants in two other studies onto the word recognition scores, i.e., the number of repeated words in the sentence. The results support the role of phonological deficits (non-native consonant perception in that specific case) in SPIN recognition. However, the findings of the study are not sufficient to evaluate the role of non-native phonological perception as opposed to top–down use of context predictability, since the sentences used in the study had the lowest cloze probabilities possible.

No data are yet available on heritage processing of SPIN. Is SPIN perception in HSs on the par with native speakers because they have the advantage of early starters, or is it degraded as in L2 learners because their proficiency is comparable to late L2 learners? While there is robust evidence that non-native speech perception is affected by noisy conditions to a greater extent than native perception, there is no agreement regarding the relative role of several factors implicated in L2 learners’ perceptual problems when processing SPIN. These factors include phonological deficits, reduced lexical knowledge, and a reduced ability to rely on top–down processing and to use contextual cues for sentence integration. The current study fills the gap and compares the perception of Russian speech in multi-talker babble noise in HSs of Russian and late L2 learners at the same proficiency levels to that of native Russian speakers. HSs of Russian in the study are early interrupted learners whose first language spoken at home was Russian, but who later switched to English, currently their dominant language. Given that heritage language is shaped by early naturalistic exposure from birth that relies exclusively on the aural modality, at least in the first years of life, one can hypothesize that HSs would have a processing advantage for SPIN over late L2 learners. Indeed, late learners, college-level students, mainly acquire Russian in a formal classroom and rely heavily on visual input, i.e., reading. While the goal of a modern foreign language classroom is to develop all four skills—two receptive, reading and listening, and two productive, speaking and writing (Rogers, 2014)—an objective assessment of the listening skills in late learners of Russian as a foreign language produced disappointing results (Thompson, 2000, p. 276). If a heritage SPIN advantage were to be found, the question arises as to the factors underlying this advantage.

### THE CURRENT STUDY

This study investigates the role of sentence context predictability and uses two levels of multi-talker babble noise, high and low, to determine whether the efficiency of processing SPIN depends on bottom–up acoustic-phonetic and/or top–down semantic-syntactic sentence integration. It goes on to compare the outcomes of the SPIN test with three additional tests of phonological and lexical knowledge in the same groups of participants^1^. To control for the role of possible phonological deficits leading to problems with efficient processing of acoustically degraded speech, the study uses two independent measures of phonological perception. Both measures target the phonological contrast that causes most difficulties for speakers of English, the hard/soft consonant contrast. The AXB discrimination task measures sensitivity to the contrast in nonsense syllables, while the picture-word discrimination task looks at the sensitivity to the same contrasts in minimal pairs of lexical items and thus investigates the robustness of phonolexical representations differentiated by the same hard/soft contrast. In order to explore the possibility that the advantage on the SPIN task may stem from superior knowledge of vocabulary, the study compares the accuracy scores on a multiple-choice task measuring vocabulary in different frequency ranges.

The study addresses the following questions:

• Are HSs as efficient as L1 speakers in listening to SPIN or do they experience the same deficits as late L2 learners at the same proficiency levels?
• Which factors are responsible for the problems experienced by HSs and L2 learners when processing SPIN: phonological deficits, lack of vocabulary knowledge, and/or the ability to rely on top–down processing and use sentence cues?
• What is the role of proficiency and learning background, early versus late start in the ability to rely on top–down processing?

## EXPERIMENT 1: SPEECH IN NOISE

### MATERIAL AND METHODS

The present study uses the design of the original SPIN test (Kalikow et al., 1977), with high- and low-probability sentences presented in two levels of noise, high and low, and the task for the participant was to repeat the last word of the sentence. It used balanced lists of words created based on a comprehensive study of Russian speech recognition in white noise, that has identified numerous factors that influence speech comprehensibility in both native and non-native speakers (Shtern, 1992). These factors form a hierarchical structure and depend on the type of stimuli: syllables, words, sentences, and extended text. Since the task in the current experiment elicits the responses at the word level, only the findings about this level are provided below. Shtern obtained the following hierarchy of factors at the word level (words presented in isolation) in native speakers that are relevant to the present study:

(1) Length of the word in phonemes: the longer the word, the better it is perceived.
(2) Part of speech: nouns are best, and verbs worst, in intelligibility.
(3) Stressed vowel: /a-o-e-i/ have better intelligibility than /u--i/^2^.
(4) Consonantal load: the more consonants in a word, the better its perception.
(5) Place of stress: disyllabic words with stress on the first syllable are perceived better than those with stress on the second syllable.

The same study emphasized that the level of predictability, defined and measured by the presence and number of key words suggesting the use of the target word, plays an important role in speech intelligibility at the sentence level and above and interacts with the level of noise and purely phonetic factors described above at the word level. Shtern (2001) created balanced word lists in such a way, that each list of 10 nouns in the Nominative case (the citation form in Russian) has the same parameters that have been demonstrated to be critical for recognition of SPIN by native Russian speakers. The lists of nouns created by Shtern and used in this study are balanced in frequency (with four gradations; only relatively high-frequency words are used), length in syllables (two monosyllabic, four disyllabic, and four trisyllabic words), stress placement, stressed vowel (two of each vowel: /a/, /u/, /e/, and /o/, and one of each: /ɨ/ and /i/), and the percentage of voiceless consonants (40–50% per list). We used eight lists with 10 nouns each to create 80 sentences.

#### Materials

The critical design of the SPIN used in this study crosses two factors: noise level and predictability of the final word based on the sentence context. In general, it is expected that higher noise levels will produce more errors. However, as proficiency increases, learners’ perception should be more robust in the face of noise, because of a greater internalization of syntactic structure, semantic properties, collocational tendencies, phonological information, etc. Therefore, sentence context was manipulated to be either highly predictive of the final word (e.g., ‘I don’t have a sister, but I have a brother’), or not at all predictive (e.g., ‘The man in the park has a brother’). It is expected that under very noisy conditions, advanced and near-native learners will show a large effect of context, where the words in highly predictive sentences are easier than the words in poorly predictive sentences. It is expected that this advantage of context will correlate with proficiency.

The task uses four conditions, with two levels of noise and two levels of context cloze probability. The high-noise level is combined with 20 high and 20 low cloze probability sentences. Identically, the low-noise level is combined with 20 high and 20 low cloze probability sentences. Thus, the task includes eight blocks of 10 sentences each—four high-probability (40 sentences), and four low-probability (40 sentences). The target word is a sentence-final noun. For the sentence-final word, the task uses phonetically balanced lists of nouns (Shtern, 2001). The carrier sentences, both high- and low-probability, were balanced for number of words (average 4.8 to 5.4 words depending on the block), and number of syllables (10.03 to 10.12 syllables). A total of 80 sentences were used. All participants listened to the same set of sentences, which made it possible to reduce the number of participants in the study and to ensure that no uneven distribution of participants with varying proficiency across different presentation lists takes place. This was imperative given that heritage and L2 participants were in the same proficiency range based on the standardized test of oral proficiency (see Participants). Sample items (2a,b) are provided below:

(2a) High cloze probability context

   U menja net sestry, no est’ brat.

   At me no sister but (there) is brother.

   ‘I don’t have a sister, but I have a brother.’

(2b) Low cloze probability context

   Rebjonok ne znal, chto eto otvet.

   Child not knew that this (was) answer.

   ‘The child did not know that this was the answer.’

Two voices, male and female, were used to record the stimulus sentences. Half of the sentences (40) were presented in the male voice, and another half in the female. Voices were not alternated, but presented in two blocks, first the male and then the female. The recordings were rescaled so that they had similar energy values. The multi-talker babble noise was produced by forward-superimposing multiple stimulus sentences from the same task so that the noise had a speech-shaped quality and the same frequency spectrum as the stimulus sentences. The level of the resulting noise was manipulated to create two noise conditions: low-noise and high-noise. The sentences were then combined with each of the two masker noise types such that the noise signal started on average 1.5 s before the onset of the sentence and continued for about 1.5 s after the sentence offset. The speech-to-noise ratio (SNR) for the low-noise condition was on average 4 dB, and the SNR for the high-noise condition was on average 1.5 dB. To determine the appropriate SNR for each sentence in the high- and low-noise conditions, a subjective piloting was used with four native speakers of Russian who did not take part in the experiment. Only sentences with the low-predictability context were used to establish the target noise level. In the high-noise condition, half of native listeners identified the last word in the sentence, while in the low-noise condition, three out of four did. Thus, the choice of the SNR for both noise conditions reflected average discriminability by native speakers of Russian established prior to the main experiment.

#### Participants

Sixty-eight people participated in the SPIN experiment and were paid for their participation. Specifically, the data were collected from 11 native speakers of Russian, 23 HSs, and 34 late L2 learners of Russian. The sample contained 31 males and 37 females. As seen in **Table 1**, the average age of the L2-high group is higher than that of the other participants, and L2 learners tend to be older on average. This tendency is understandable given that it takes several years to reach the low-level Russian proficiency threshold established in this study, and even longer to achieve very high proficiency. Given that the experiment did not collect reaction time data, these age differences are not expected to bias the results. The SPIN test was part of a larger 4-h long test battery (Gor and Cook, 2010; Long et al., 2012), and the results of the SPIN test are compared below to the tests gaging phonological discrimination and vocabulary control in the same heritage and L2 participant groups. HSs who participated in this experiment had Russian-speaking parents, were exposed to Russian from birth and heard it spoken at home on a daily basis. However, they had lived in the U.S. since the age of 7 on average (range: 0–14), and considered English to be their dominant language, and Russian, the language of the test, their weaker language. HSs did not live in Russia or a Russian-speaking country after puberty, and had little or no formal elementary schooling in the Russian language, although all of them could read in Russian. Late L2 learners were all native speakers of American English and started learning Russian after puberty in a formal classroom, most of them as young adults in college. The average age of onset of Russian was 18.4 years (range: 13–27), and an average length of formal study was 10 years (range: 0–39). While all but five L2 learners had a study abroad experience in Russia or a Russian-speaking country, they did not learn Russian in a naturalistic setting, merely by virtue of living in a Russian-speaking country or community.

**Table 1:** Background information of the participants in the study.

Participant group	*N*	Age mean (range)	Gender M/F
Native speakers	11	25.55 (22–30)	3/8
Heritage speakers, high proficiency	12	24.08 (18–51)	4/8
Heritage speakers, low proficiency	11	20.81 (18–25)	3/8
L2 learners, high proficiency	18	41 (25–56)	12/6
L2 learners, low proficiency	16	28 (21–44)	8/8
Total	68	29.31 (18–56)	31/37

*High proficiency includes oral proficiency levels 2+, 3, 3+, 4; low proficiency includes oral proficiency levels 1, 1+, 2 based on the Interagency Language Roundtable (ILR) oral proficiency scale.*

Heritage speakers and L2 learners of Russian in this experiment were divided into two groups, high- and low-proficiency, using the Interagency Language Roundtable (ILR) testing format, which made possible direct comparisons of the high- and low-level proficiency heritage and L2 participants (Long et al., 2012)^3^. The ILR score is established based on an audio-recorded oral proficiency interview conducted with a certified tester. The interview lasts 20–30 min and takes the form of a rigidly structured conversation, although the topics of the conversation vary depending on the testee’s background. The ILR oral proficiency score is a standard global language proficiency score widely accepted in the U.S. In addition to the base levels, the ILR scale has “plus” sublevels that refer to the proficiency exceeding the requirements of the level. In our participant groups, both heritage and L2, the oral proficiency scores ranged from 1 (Intermediate) to 2 (Advanced), 3 (Superior), and 4 (Distinguished). Both the heritage and L2 samples also included “plus” sublevels, e.g., 1+ (Intermediate High). The participants were divided into low-proficiency groups containing participants with the ILR scores ranging from 1 to 2 (16 L2 and 11 HSs), and high-proficiency groups containing participants with ILR scores ranging from 2+ to 4 (18 L2 and 12 HSs). A detailed breakdown by age, gender, and proficiency level is provided in **Table 1**.

#### Procedure

The listening materials in the SPIN task were presented in two blocks of 40 sentences, the first recorded in a male voice and the second in a female voice, with a short pause between the blocks. Each set of 40 sentences included all four critical conditions, high-noise/high-predictability context, high-noise/low-predictability context, low-noise/high-predictability context, and low-noise/low-predictability context. The order of the sentences in these four conditions was randomized within each block (male-voice and female-voice), and was the same for all participants. Participants were tested individually, and were seated in a quiet room in front of *Dell® Latitude/D820* computers with *Plantronics*. Audio 750 headsets with mounted microphones and *Logitech® Precision USB* game pads. They were presented with instructions on the computer screen in English, and used buttons on their game-pad to initiate the following trial. Participants listened to the entire sentence in noise and were then asked to repeat the sentence-final word into the microphone. The experiment was self-paced and took ~20 min. Participants were encouraged to take a break in the middle. All four experiments reported in the present publication were part of a larger test battery and were completed on the same day. Ample rest time was provided to participants to reduce possible fatigue. Also, the type of activity varied from one task to the next, which lessened the effect of monotony. The experiment was programmed in DMDX (Forster and Forster, 2003). Responses were recorded and then manually transcribed by trained linguists, native speakers of Russian. No substitutions were accepted when scoring the responses for accuracy. Only correct responses were scored as 1; all the other responses, e.g., responses with a phonological neighbor, were coded as 0. The accuracy score results were subjected to statistical analyses.

### RESULTS

**FIGURE 1:**
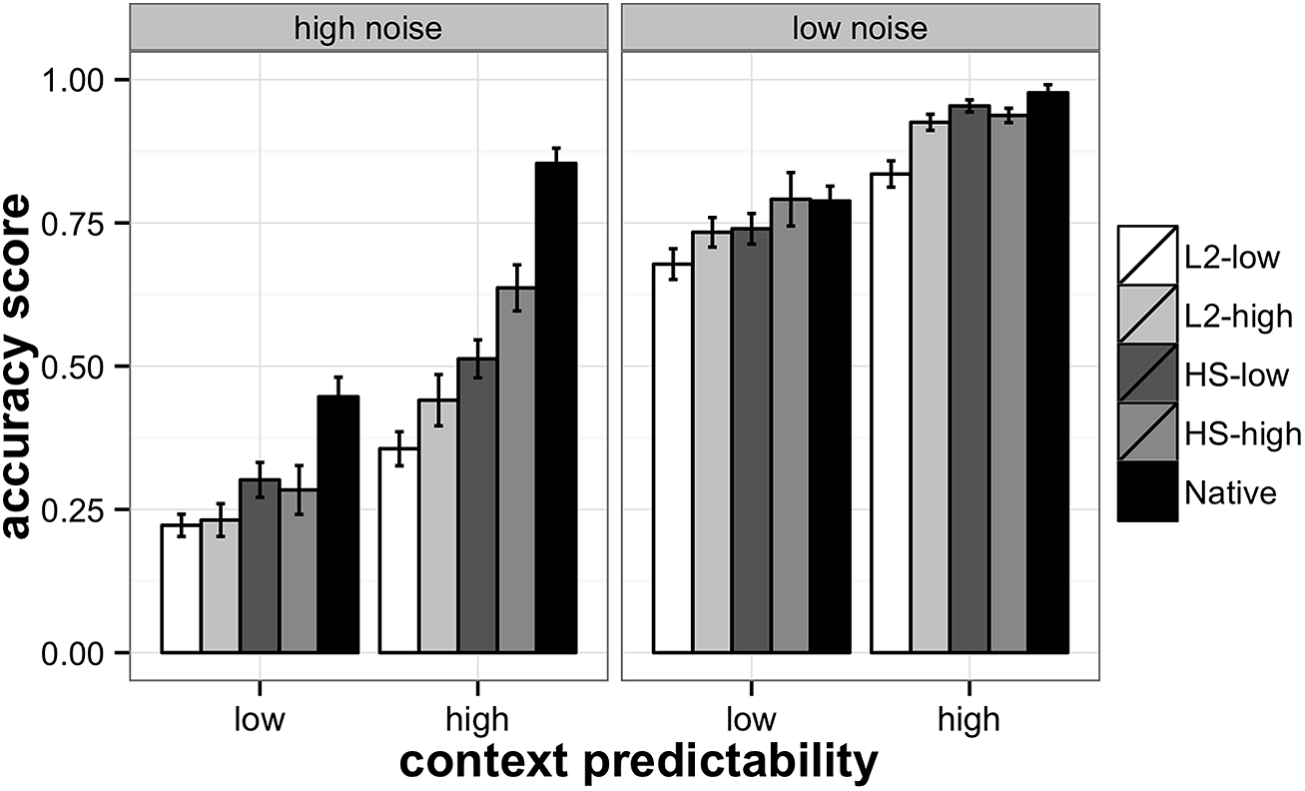
**Accuracy scores on SPIN task in heritage, L2, and native participants.** Heritage and L2 participants are divided into high- and low-proficiency groups. The left panel represents the high-noise and the right the low-noise conditions. L2-low – low-proficiency L2 learners, L2-high – high-proficiency L2 learners, HS-low – low-proficiency heritage speakers, HS-high – high-proficiency heritage speakers, and Native – native speakers of Russian.

The accuracy scores for each participant group broken down by the level of noise and context predictability are presented in **Table 2** and **Figure 1**. Participants’ responses were analyzed with a repeated measures ANOVA in by-subject and by-item analyses. The study had a 2 × 2 × 5 factorial design, with the following predictor variables: context predictability (two levels: low and high), noise level (two levels: low and high), and language proficiency group (five levels: L2-low, L2-high, HS-low, HS-high, Native). The dependent variable was the accuracy of correctly identified words in a sentence. R statistical package was used for the analyses (R Core Team, 2013, version R 3.01). The results are represented in **Table 3** (by-subject) and **Table 4** (by-item).

**Table 2:** Participants’ mean accuracy scores across all conditions.

	Language group				
	L2-low	L2-high	HS-low	HS-high	Native
HN/HC	0.36	0.44	0.51	0.64	0.85
HN/LC	0.22	0.23	0.30	0.28	0.45
LN/HC	0.84	0.93	0.95	0.94	0.98
LN/LC	0.68	0.73	0.74	0.79	0.79

*HN, high noise; LN, low noise; HC, high context predictability; LC, low context predictability.*

**Table 3:** Repeated measures ANOVA results for Experiment 1: speech in noise, by-subject analyses.

By-subject

	**Df**	**Sum Sq**	**Mean Sq**	***F*** **value**	**Pr(>F)**
**Between-subject**					
Group	4	1.767	0.4417	22.29	<0.000
Residuals	63	1.249	0.0198		
**Within-subject**					
Context type	1	3.089	3.089	322.633	<0.000
Noise	1	12.013	12.013	1254.523	<0.000
Context type:Noise	1	0.082	0.082	8.61	0.00376
Context type:Group	4	0.176	0.044	4.585	0.00148
Noise:Group	4	0.525	0.131	13.707	<0.000
Context type:Noise:Group	4	0.18	0.045	4.7	0.00122
Residuals	189	1.81	0.01		

**Table 4:** Repeated measures ANOVA results for Experiment 1: speech in noise, by-item analyses.

By-item

	**Df**	**Sum Sq**	**Mean Sq**	***F*** **value**	**Pr(>F)**
**Between-item**					
Context type	1	9.748	9.748	31.9	<0.000
Residuals	78	23.833	0.306		
**Within-item**					
Noise	1	32.95	32.95	1044.759	<0.000
Group	4	5.23	1.31	41.465	<0.000
Context type:Noise	1	0.22	0.22	7.087	0.00794
Context type:Group	4	0.58	0.15	4.602	0.00113
Noise:Group	4	1.71	0.43	13.584	<0.000
Context type:Noise:Group	4	0.44	0.11	3.509	0.00756
Residuals	702	22.14	0.03		

The analysis revealed a significant context effect indicating that participants on average performed better in the high-predictability context condition. A significant noise effect suggests that word identification was significantly more accurate in the low-noise condition, and a language group effect supports the differences among the participant groups. There were also significant context by noise, context by group, and noise by group two-way interactions. Finally, a three-way interaction between context, noise and language group was also found significant, suggesting that the interaction between noise and context changed across the levels of the language group variable. Separate ANOVAs for each group showed that two-way interactions between context and noise were significant in the Native [*F_1_*(1,252) = 10.93, *p* < 0.01; *F_2_*(1,2215) = 3.96, *p* = 0.05], and the HS-high [*F_1_*(1,252) = 10.64, *p* < 0.01; *F_2_*(1,2215) = 9.85, *p* = 0.01], groups, while they were statistically insignificant in the L2-low, L2-high, and HS-low groups.

**FIGURE 2:**
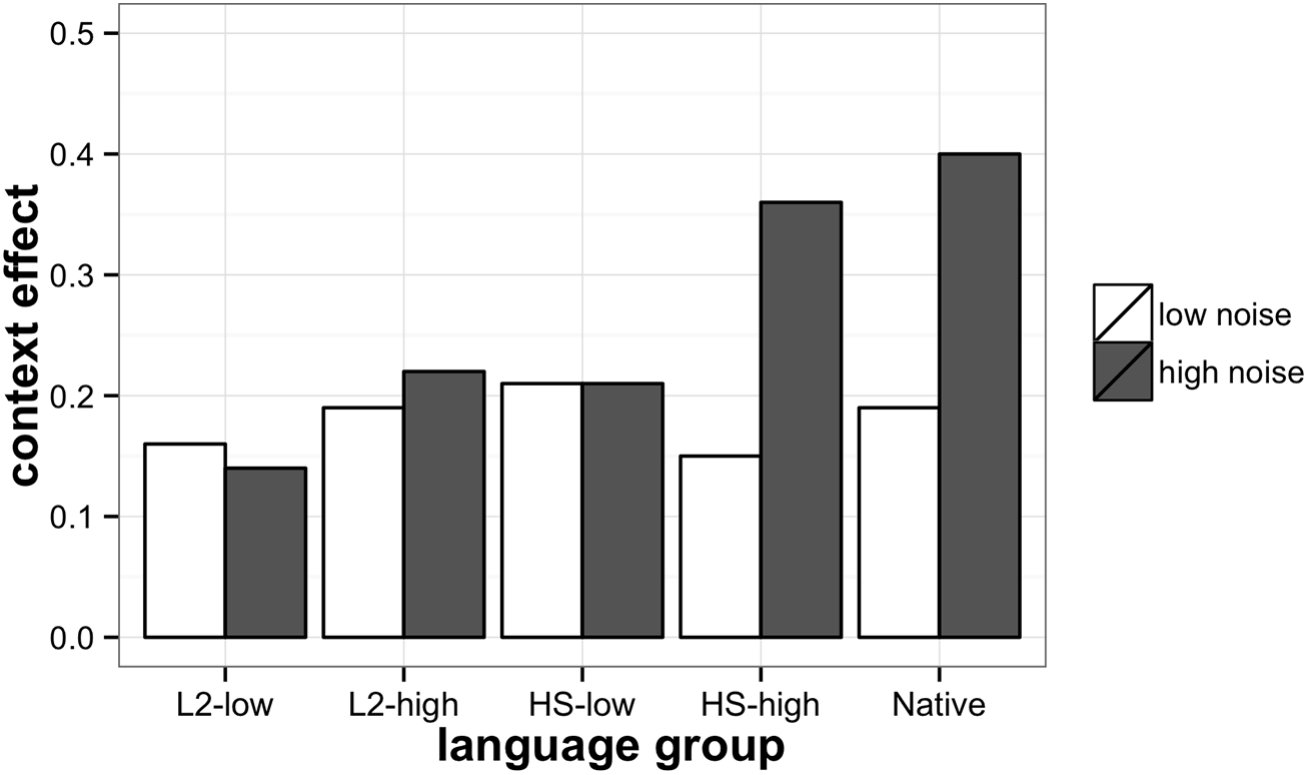
**Context effects in SPIN task for heritage, L2, and native participants.** Context effect is calculated as a difference between the score in the high-predictability context condition and low-predictability context condition. 0.5 corresponds to 50% increase in accuracy in the high-predictability condition.

These data are represented visually in **Figure 2** where the difference between the accuracy score in the high-predictability and low-predictability conditions is provided as a percentage. This difference accounts for the context effects on response accuracy under the same noise levels.

**Figure 2** demonstrates that while L2-low, L2-high, and HS-low groups benefited from high context predictability to a similar extent regardless of the noise condition (low or high), Native and HS-high groups appear to rely on context to a greater extent (almost 40% more) when they listen to sentences in high-noise compared to low-noise, or in other words, they take advantage of the context when it is both needed and available. TukeyHSD *post hoc* tests showed that the increasing group differences (from L2-low to Native group) in the accuracy scores in the high-noise/high context condition were significant across all group comparisons (*p* < 0.5) except for between L2-low and L2-high [*t*(63) = –1.58, *p* = 0.13], L2-high and HS-low [*t*(63) = 1.29, *p* = 0.2]. In the high-noise/low context condition, the differences were significant between Native and other language groups [L2-low: *t*(63) = –5.72, *p* < 0.001, L2-high: *t*(63) = –4.84, *p* < 0.001, HS-low: *t*(63) = –3.17, *p* < 0.01, HS-high: *t*(63) = –2.9, *p* < 0.01].

To summarize, predictably, all groups benefited from low-noise compared to high-noise, however, the role of context predictability depended on the participant group and interacted with the level of noise. In the low-noise condition, there was no need in the context to recover the sentence-final word, while in the high-noise condition, the ability to efficiently process the context and to generate predictions that would help to recover the acoustically degraded sentence-final word was crucial for performance. According to the obtained results, only two groups were able to take advantage of the high-predictability context in the high-noise condition, native speakers and HSs in the high-proficiency group. These two groups relied on the context significantly more in the high-noise than in the low-noise condition. All the other groups, low-proficiency L2 and heritage, and high-proficiency L2, improved their SPIN recognition due to the high-predictability context at both noise levels to a similar, limited extent. Obviously, the high-noise/high-context condition was critical for exploring the differences among the groups, because the context was available, but the high level of noise simultaneously made the use of the context difficult. Group comparisons of accuracy scores in the critical high-noise/high-context condition reveal that native speakers are more accurate in processing SPIN than all of the other groups, and at each proficiency level, high and low, HSs outperformed L2 learners, with the L2 high-proficiency group performing similarly to the heritage low-proficiency group. Thus, HSs showed an advantage over late L2 learners, but a disadvantage compared to native speakers.

A question arises as to what deficits underlie the non-native disadvantage in late L2 learners and what aspect of SPIN processing creates advantages for HSs compared to L2 learners. In the next sections, we will briefly report the results of three experiments targeting phonological and lexical control in the same groups of participants. We will then discuss the patterns observed in the non-native populations in relation to their language learning background and setting. Two experiments tested the heritage and L2 participants’ sensitivity to the phonological hardness/softness contrast that is very prominent in Russian, as it differentiates 12 pairs of Russian consonants and is widely used contrastively in building the sound shape of words and morphemes. For example, Russian infinitives and third person singular non-past tense for many verbs is contrasted by the hardness/softness of the final consonant, e.g., /pomn’it’/^4^ means ‘remember’ while /pomn’it/ means ‘he/she remembers,’ with the last consonant, soft /t’/or hard /t/, providing the phonological shape for this morphosyntactically loaded contrast (Chrabaszcz and Gor, 2014). The first experiment, AXB discrimination^5^, targeted lower-level perceptual sensitivity to the phonological hard/soft contrast, while the second, Picture-Word Discrimination, tested phonolexical representations, or representations of words as phonemic sequences.

## EXPERIMENT 2: AXB DISCRIMINATION

### MATERIAL AND METHODS

#### Materials

AXB discrimination test targeted the hard/soft phonological contrast in Russian consonants that has been shown to present perceptual difficulties for late American learners of Russian (Lukyanchenko and Gor, 2011; Chrabaszcz and Gor, 2014). This contrast involves the whole consonantal system of Russian with most (but not all) consonants paired according to the hard/soft feature. The hard/soft contrast is absent in English, and accordingly, English-speaking learners of Russian are not sensitive to this contrast. The test items included 84 monosyllabic consonant-vowel-consonant (CVC) non-words involving the Russian hard-soft consonant opposition (a total of 168 tokens). The stimuli were recorded by five native speakers of Russian, two males, and three females. Multi-talker speech samples ensured that the listeners would not be guided by lower-level acoustic properties of the stimuli rather than the phonological contrasts. Participants needed to process phonologically same CVC stimuli in the pronunciation of different speakers at the phonological level to establish a phonological equivalence. The study used three conditions: (1) /t-t’/ in the word-final position, as in /dot – dot’/, (2) /p-p’/ in the word-final position, as in /dop – dop’/, and (3) the /C’V-CjV/ condition, where a soft consonant in the word-initial position was contrasted with a combination of a hard consonant with a palatal /j/, as in /m’a – mja/. The contrasts and positions were selected based on the literature (Kochetov, 2002; Bondarko, 2005), and our previous research (Lukyanchenko and Gor, 2011; Chrabaszcz and Gor, 2014), as presenting the most perceptual difficulty for non-native listeners. All the available data converged on the fact that the word-final position was perceptually the most difficult one, and that the /t-t’/ contrast was easier than the /p-p’/ contrast. This is due to the fricativization of the soft /t’/ that provides a perceptual cue to the soft feature. There were 28 contrasts in each condition, and all contrasting consonants occurred in various vowel environments.

In order to control for the position of each token, the contrasts were grouped into triplets of four different kinds, AAB, ABB, BBA, and BAA (e.g., /mit – mit – mit’/, /mit – mit’ – mit’/, etc.). The A and B items differed by the hard and soft consonants in the word-final position, and by the /C’V-CjV/ contrast in the third experimental condition. The critical token, X, always occurred in the middle. In half of the trials X corresponded to A, and in half of the trials to B. Each triplet consisted of different recordings by different speakers, and was never a repetition of the same recording by the same speaker.

#### Participants

The participants in AXB discrimination test were the same as in the SPIN test.

#### Procedure

Participants were auditorily presented with three stimuli (A, X, and B), separated by an interval of 335 ms. They were told that the first segment (A) was always different from the third segment (B), and that their task was to decide whether the second segment (X) should be categorized as A or B. Participants were required to press one of two buttons on the gamepad, left or right, to indicate whether X was identical to A or to B respectively. The next trial started 835 ms after each response. No feedback was provided. The DMDX software platform was used for stimuli presentation. The experiment took 10 min to complete.

### RESULTS

**FIGURE 3:**
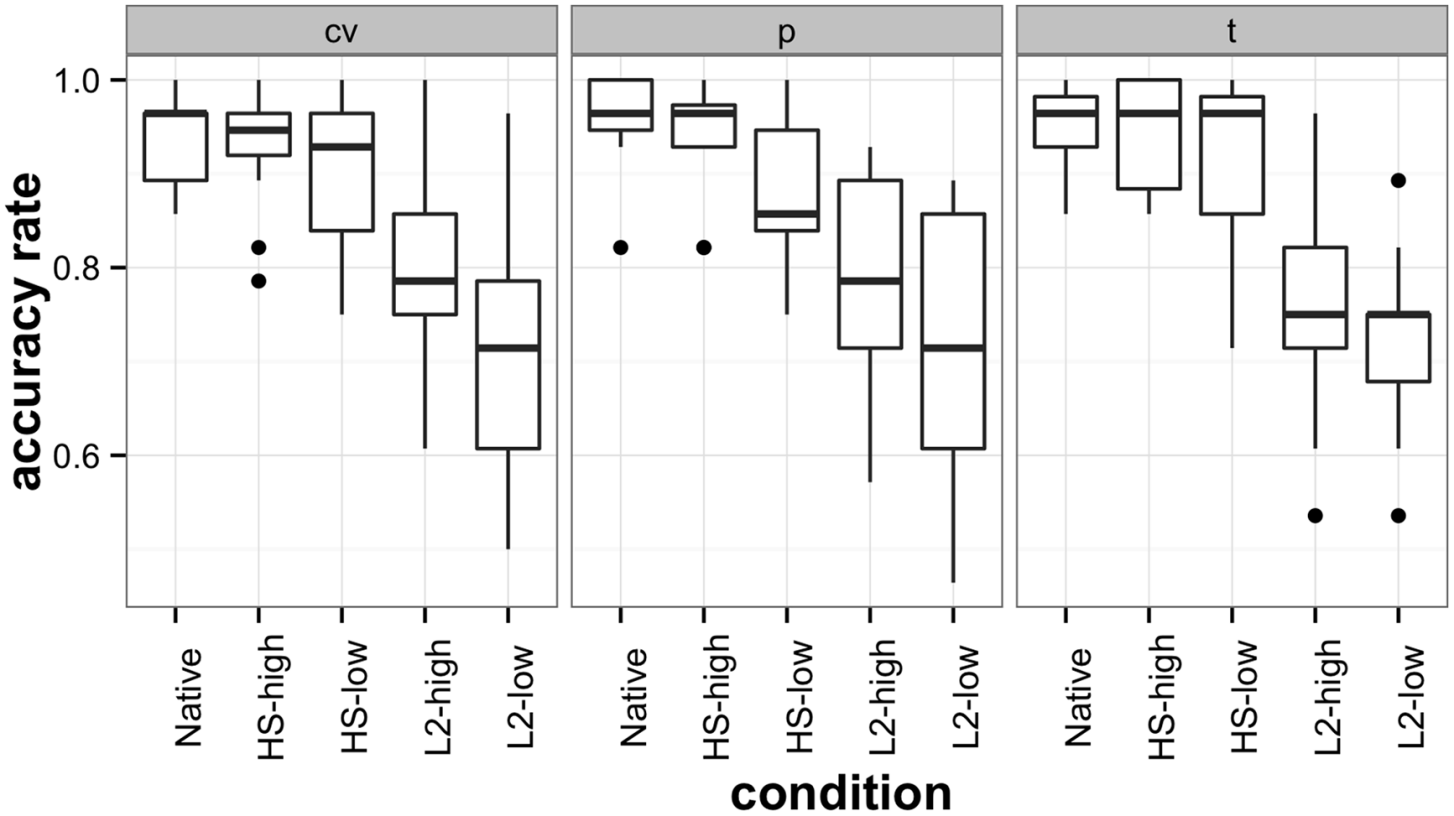
**AXB discrimination task accuracy scores across all groups.** The labels ‘cv’, ‘p’, and ‘t’ represent the three critical conditions, the C’VC-CjVC, final /p–p’/, and /t–t’/ consonants.

A split-plot analysis of variance was used to compare the mean accuracy scores of native, heritage, and L2 speakers of Russian on the three types of contrasts: /t-t’/, /p-p’/ and /C’V-CjV/ contrast. Using an alpha level of 0.05, the results yielded a significant interaction between language group and contrast type [*F*(7.91,3759.36) = 6.2, *p* < 0.01]^6^, a significant within-subjects main effect of contrast type [*F*(1.98,3759.36) = 24.82, *p* < 0.01], and a significant between-subjects main effect of language group [*F*(4,1899) = 91.59, *p* < 0.01]. The effects are represented graphically in **Figure 3**. With regard to the HSs’ performance, pairwise *t*-tests indicated that while the accuracy rate of the high-proficiency HS group was not statistically different from that of the native group [*t*(63) = 1.03, *p* = 0.3], the low-proficiency group performed significantly less accurately than the native group [*t*(63) = 3.25, *p* < 0.005]. Both L2 groups, high- and low-proficiency, were statistically less accurate than both HS groups [L2-low – HS-low: *t*(63) = –7.83, *p* < 0.001, L2-low – HS-high: *t*(63) = –11.39, *p* < 0.001, L2-high – HS-low: *t*(63) = –5, *p* < 0.001, L2-high – HS-high: *t*(63) = –8.2, *p* < 0.001] and also significantly different from each other [L2-low – L2-high: *t*(63) = 2.87, *p* < 0.01], with the low-proficiency L2 group performing the least accurately of all of the language groups.

Thus, the AXB discrimination task demonstrated the consistent advantage of proficiency-matched heritage participants compared to late L2 learners in phonological discrimination of non-word segments that had no lexical representations in the mental lexicon. Moreover, high-proficiency HSs’ sensitivity to the hard/soft contrast did not differ statistically from that native speakers’. At the same time, native speakers outperformed low-proficiency HSs on all three contrasts involving hard/soft consonants.

## EXPERIMENT 3: PICTURE-WORD DISCRIMINATION

The motivation behind the picture-word discrimination task was to investigate phonological processing of HSs and L2 learners of Russian in words, thereby testing the robustness of phonolexical representations. The task used minimal pairs of words, with accurate spoken word recognition depending on the discriminability of the same hard/soft contrast that was used in the AXB discrimination task. The task examined how the two populations of non-native listeners perform the mapping of the auditory input on to the stored phonological-lexical template of the word, and how their performance is similar to or different from that of native speakers of Russian.

### MATERIAL AND METHODS

#### Materials

The stimulus materials for the task were divided into two conditions, a critical condition and a control condition. The critical condition included twelve minimal word pairs that can be distinguished based on the hardness or softness of the consonant, e.g., /mat/ ‘checkmate,’ versus /mat’/ ‘mother.’ Twelve minimal pairs were constructed for the control condition, which differed from each other in consonant voicing based on the distinction between voiced and voiceless consonants, as in /do
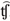
ka/ ‘daughter,’ versus /to
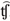
ka/ ‘period/period,’)^7^. Additionally, four distractor minimal word pairs were constructed, and two practice items were added at the beginning of the task. The materials included 30 minimal pairs (60 words). The words were mixed randomly and were recorded by a male native speaker of Russian. The words were counterbalanced between two presentation lists in such a way that both words from the same minimal pair did not occur within the same list. The same professional artist drew all 60 pictures depicting the words, so that potential differences in their visual salience would not create any biases. Lexical frequency of the words constituting the minimal pairs was not controlled, because only a few minimal pairs of nouns referring to entities that can be represented by an easily recognizable picture and differentiated by the hard/soft consonant contrast are available in Russian. However, all the words were in the high to medium frequency range.

#### Participants

The participants in the picture-word discrimination task were the same as in two foregoing tasks, SPIN and AXB discrimination.

#### Procedure

Participants heard one word from the minimal pair followed by two pictures appearing on the computer screen. The presentation of the stimuli was controlled by DMDX software with the gamepad used for input. The test-takers had to decide which of the two pictures correctly matched the word that they just heard. The correct picture appeared on the left side of the screen in half of the trials, and on the right side in the other half. The participants were instructed to use the left trigger button on the gamepad to select the picture on the left, and the right trigger button to select the picture on the right. Feedback was only provided during the practice session in the beginning. The experiment took 5 min. Participants received a score of 1 selected for each test item where they selected the correct picture, otherwise they received a score of zero for the item. The accuracy scores were used for data analysis.

### RESULTS

A repeated measures analysis of variance was used to compare the mean accuracy scores of native speakers (*M* = 0.99, SD = 0.09), high-proficiency HSs (*M* = 0.98, SD = 0.14), low-proficiency HSs (*M* = 0.79, SD = 0.41), high-proficiency L2 speakers (*M* = 0.76, SD = 0.42), and low-proficiency L2 speakers of Russian (*M* = 0.60, SD = 0.49)^8^. The results for both by-subject and by-item analyses were significant and are reported in **Table 5**.

**Table 5:** Repeated measures ANOVA results for Experiment 3: picture-word discrimination.

	Df	Sum Sq	Mean Sq	*F* value	Pr( > F)
**By-subject analysis**					
Proficiency level	4	1.4355	0.3589	27.29	<0.000
Residuals	62	0.8153	0.0132		
**By-item analysis**					
Proficiency level	4	1.2704	0.3176	29.14	<0.000
Residuals	44	0.4795	0.0109		

**FIGURE 4:**
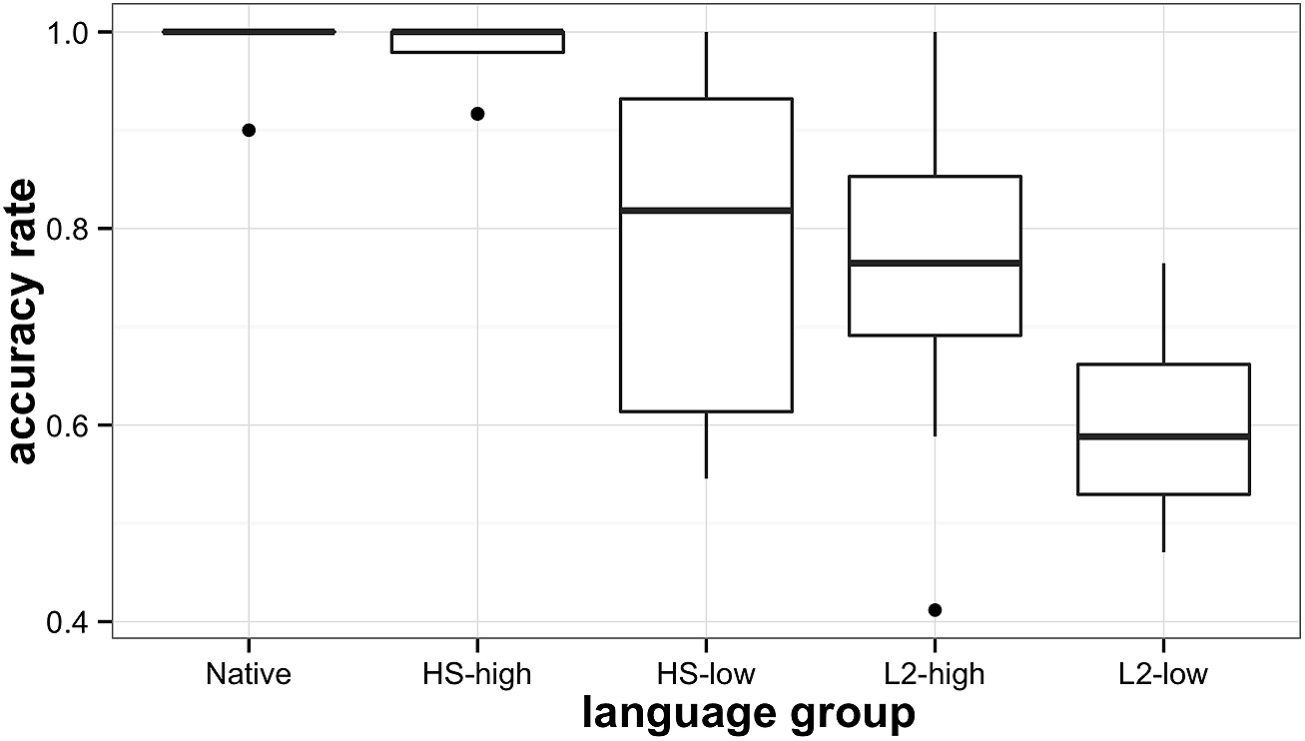
**Heritage, L2, and native participants’ accuracy scores on picture-word discrimination task**.

Pairwise *t*-tests indicated that the native speakers of Russian and high-proficiency HSs of Russian were not statistically different from each other [*t*(63) = 0.73, *p* = 0.47], but were significantly different from the remaining language groups. Low-proficiency HSs performed similarly to high-proficiency L2 speakers [*t*(63) = 0.38, *p* = 0.7], and both groups were significantly more accurate in their responses than the low-proficiency L2 group [HS-low: *t*(63) = 3.4, *p* < 0.05, L2-high: *t*(63) = 3.68, *p* < 0.01, respectively]. The results are displayed graphically in **Figure 4**.

To summarize, as in the AXB discrimination task, HSs outperformed proficiency-matched late L2 learners in picture-word discrimination that involves matching the auditory input to stored phonolexical representations of words. At the same time, only the high-proficiency heritage group approximated native speakers’ accuracy scores. Thus, overall, HSs show an advantage in phonological sensitivity both in a non-word task, and in a task involving phonolexical representations of stored words. In both tasks, only the high-proficiency heritage group’s accuracy scores matched native speakers’ scores.

## EXPERIMENT 4: LEXICAL KNOWLEDGE MULTIPLE-CHOICE TRANSLATION TEST

### MATERIAL AND METHODS

When listening to sentences with a highly predictable context, listeners integrate the incoming information with the parts of the sentence that have already been processed and develop expectations about the upcoming word (Zwitserlood, 1989; Laszlo and Federmeier, 2009). The ability to develop predictions helps listeners to process the sentence-final word in high levels of noise. Given that the whole sentence is degraded due to noise, a non-native listener needs to be resistant to acoustically and phonetically degraded speech, be fast and efficient at vocabulary retrieval, and be able to quickly and efficiently generate expectations about upcoming words, integrate these expectations with what has been heard, and continue generating expectations (Mayo et al., 1997; van Wijngaarden et al., 2002; Bradlow and Alexander, 2007; Cervera and González-Alvarez, 2011). The lexical knowledge test compares vocabulary knowledge in HSs and L2 learners to determine whether the HSs’ advantage in SPIN could be explained by their superior vocabulary knowledge.

#### Materials

The materials consisted of words in three lemma frequency ranges as determined based on Sharoff’s Corpus (Russian online corpus, approximately 90 million words at the time of use^9^) that later became part of the Russian National Corpus^10^. The frequency ranges were chosen to approximate the frequencies used in a study of the M350 component, a neural response to lexical frequency (Embick et al., 2001): high-frequency, average 140 ipm (range 130–170), medium-frequency 30 ipm (30–60), and low-frequency 6 ipm (6–7). The numerical values correspond to the number of items per 1 million words of running text in the corpus (items per million, ipm). The task included nouns (*N* = 30, 15 concrete and 15 abstract), adjectives (*N* = 15), and verbs (*N* = 50), and since the target words in SPIN task were nouns, the results for this task will be briefly summarized.

#### Participants

The participants in the lexical knowledge multiple-choice translation test were the same as in three foregoing tasks.

#### Procedure

The lexical knowledge test is a multiple-choice auditory task with the Russian word presented auditorily, and three translation options in English presented visually on the computer screen. Participants were asked to choose the correct option by pressing the corresponding button on the keyboard, and their responses were recorded electronically. The experiment was run using DMDX software and took 10 min to complete.

### RESULTS

Based on an ANOVA, there were no statistical differences between the participant groups, heritage and L2. Conversely, the expected differences for proficiency levels and noun frequency ranges were present. Therefore, HSs showed no advantage in lexical knowledge compared to proficiency-matched late L2 learners.

## DISCUSSION

The SPIN task presented the participants with Russian high- and low-predictability sentences in two levels of multi-talker babble noise, and compared their accuracy in repeating the last word of the sentence. Three groups of participants, native speakers of Russian, proficiency-matched HSs, and late L2 learners of Russian with American English as their dominant language took part in the experiment. The HSs and L2 learners were further divided into high- and low-proficiency groups based on their scores on a standardized test of oral proficiency, the oral proficiency interview, resulting in five groups. Results showed that only two groups, native speakers and high-proficiency HSs, took advantage of the high-probability context in the high-noise condition. Neither high-proficiency late L2 learners, nor low-proficiency participants improved their performance in the high-probability context. These findings must be interpreted on two levels. First, we must consider the potential role of different factors underlying the observed pattern of SPIN results across different groups. Second, the reported difference between HSs and late L2 learners needs to be connected to the language learning backgrounds in these two proficiency-matched groups.

Given that the study used multi-talker babble noise that combined energetic and informational masking, one can expect an impact of noise on all levels of speech processing, from low-level acoustic-phonetic interference to high-level sentence integration. Which levels were responsible for the observed differences in heritage and L2 processing of SPIN, and what features/aspects of their respective learning backgrounds could have contributed to the differences in processing degraded speech? Two experiments targeting phonological processing and one experiment testing lexical knowledge with the same participant groups as the ones that took part in the SPIN test make it possible to gage phonological and lexical knowledge of the heritage and late L2 learners and compare the outcomes to the results of the SPIN test. AXB discrimination used non-word CVC syllables and assessed participants’ sensitivity to the hard/soft phonological contrast in Russian consonants. The picture-word discrimination task tested the same contrast in minimal pairs of words and examined the robustness of phonolexical representations differentiated by this contrast. Both tasks produced the same results: HSs outperformed proficiency-matched L2 learners, and high-proficiency HSs’ accuracy scores were statistically similar to those of native speakers. HSs showed both a phonological and phonolexical advantage over L2 learners, and the high-proficiency heritage group approximated phonological sensitivity and the robustness of phonolexical representations of native speakers. Importantly, no direct statistical comparisons were made due to the differences in the design and the variables included in the three experiments. Therefore, one could argue that the comparisons between the outcomes of SPIN and the phonological tasks are suggestive rather than conclusive. The logic behind the comparisons of the global outcomes of the experiments was that both phonological tasks targeted the most difficult Russian phonological contrast for American learners, and crucially, the most pervasive one in the Russian consonantal system (Chrabaszcz and Gor, 2014). They gaged non-native sensitivity to different positional and contextual allophones, in words and non-words, and thereby provided a global assessment of phonological control. Conversely, no advantage of HSs over L2 learners was demonstrated in the lexical knowledge test, and therefore their superior lexical knowledge and/or better lexical entrenchment should be discarded as a possible explanation of the heritage advantage in SPIN.

Note that if this phonological advantage were the only cause of the differences in the SPIN results, there would have been no interaction between the level of noise and context predictability in the heritage or any other group. Conversely, two groups show a noise/context interaction, and these are native speakers and high-proficiency HSs. At the same time, the high-proficiency L2 group approximates the accuracy scores of the heritage low-proficiency group and does not show any noise/context interaction in SPIN. In other words, high-proficiency helps HSs to take advantage of the high-predictability context in the high-noise condition (as do native speakers), but the context does not help proficiency-matched late L2 learners. It is exactly this context/noise interaction with heavier reliance on the context only in high-proficiency HSs and native speakers of Russian that indicate that, indeed, superior phonological decoding abilities combined with the efficiency in quickly integrating the incoming information with the preceding sentence context and generating predictions about the upcoming word are the properties of native and high-proficiency heritage processing of speech in adverse conditions. Importantly, while HSs’ advantage over L2 learners has been documented both in SPIN and the tests of phonological sensitivity, only high-proficiency heritage listeners approximate native speech recognition in adverse conditions. In order to do so, they should be able to generate predictions by relying on efficient bottom–up and top–down mechanisms of speech processing, phonological decoding, and sentence integration that act in a bootstrapping fashion.

Heritage speakers acquire their language as native speakers, in a naturalistic environment, since birth, from their caregivers, and as their first language. This language learning background should provide them with a native advantage in auditory speech recognition demonstrated in studies of native and non-native speech perception (see Introduction). At the same time, HSs differ from native speakers and balanced bilinguals in that their proficiency in what was chronologically their first language is non-native. This is why they can be matched in proficiency with late L2 learners. If an early naturalistic start from birth always provides an advantage to HSs over late L2 learners, regardless of the proficiency level, both heritage groups should outperform both L2 groups. If language proficiency also matters, first, high-proficiency HSs will outperform low-proficiency ones, and second, there could be a proficiency-based effect that will be observed only in one proficiency range, but not the other.

The outcomes of the SPIN experiment supported both the early advantage and the role of proficiency in HSs. Thus, HSs outperformed late L2 learners, and his advantage was present both in the high-proficiency and low-proficiency-based comparisons. The results for the critical high-noise and high context predictability condition, where the differences among the groups were the most salient, show an advantage of HSs over L2 learners, thereby supporting the early heritage advantage. At the same time, the study found that the ability to profit from the high-predictability context in the high-noise condition was mitigated by proficiency in HSs. Only the high-proficiency heritage group showed native-like reliance on context probabilities in high-noise. This latter finding speaks to the role of proficiency in heritage as well as L2 listeners, given that proficiency-matched late L2 learners consistently lag behind HSs.

The reasons why HSs’ proficiency in their heritage language falls short of native proficiency are beyond the scope of this study and are still widely debated. These are predominantly the developmental factors, attrition, incomplete acquisition or, frequently, a combination of both, with their relative weight depending on the age of reduced exposure to the heritage language, and the amount of exposure to L1 and L2 since the age of L2 onset (Montrul, 2008, 2012; Bylund, 2009; Keijzer, 2010; Schmid, 2010; Polinsky, 2011). The age of L2 onset, while a decisive factor, is not the only one; the relative amount of time each of the two languages is used by a HS is no less important. Crucially, language aptitude is positively correlated with such aspects of heritage language proficiency as grammatical knowledge, as assessed by a grammaticality judgment test (Bylund et al., 2010). The study by Bylund and colleagues targeted prepubescent attriters who experienced a break with their Spanish L1 (heritage language) before puberty and switched to Swedish. In the participants with below-average aptitude, scores on the grammaticality judgment test positively correlated with the amount of daily use of Spanish. Heritage language proficiency level is thus a product of a complex interplay of cognitive, social and environmental factors. It is influenced by the amount of exposure to, and the level of engagement with, each of the two languages, which interact with language aptitude.

It should be noted that establishing language proficiency is both a theoretical and a practical problem, and the current study uses the standard of global language proficiency testing, an oral proficiency interview. It establishes the level based on the ability of the participant to perform language functions, such as being able to narrate an event in major time frames or handle a situation with a complication in a role-play situation. This format is suitable for testing HSs (see Kagan and Friedman, 2003; Polinsky and Kagan, 2007), it avoids the bias of visually presented reading materials, and since the oral proficiency interview has an interactive format, it tests both receptive (listening) and productive (speaking) skills. The assessment of the global ability to speak and interact with a conversational partner in order to establish a level of language proficiency is ecologically valid, given that language is primarily a means of communication. At the same time, the results of the study raise the issue of an asymmetry between the levels of performance on separate linguistic aspects, such as phonological sensitivity or speech recognition in adverse conditions and the global proficiency rating established in a conversational format. According to the obtained results, groups of non-native speakers with different learning profiles, but matched on speaking proficiency may still differ in their control of individual aspects of linguistic performance, a finding that needs further research. This fairly plausible finding documents the specific role of early naturalistic language exposure in shaping the mechanisms underlying phonological processing and speech recognition.

The results of the study shed new light on the role of early naturalistic experience in learning a heritage language, the first language to be learned chronologically, but the second in dominance, and characterized by non-native levels of proficiency. The reported results suggest that early naturalistic language learning experience is necessary, but insufficient for developing native listening strategies that ensure robust speech recognition in adverse conditions. Listeners encounter different forms of degraded speech in their everyday experience, ranging from band-pass filtered speech in phone communications to listening to speech in noisy conditions and separating the speech stream of the interlocutor from that of another individual speaking at the same time. It appears that a high-proficiency in the heritage language is necessary for robust speech recognition in adverse conditions. The length and intensity of exposure to, and use of the heritage language initially acquired naturalistically most likely mitigate the high or low heritage language proficiency level. To the best of our knowledge, this is the first study devoted to speech recognition under adverse conditions in HSs. It has established that high-proficiency HSs outperform oral proficiency-matched late L2 learners on the use of contextual information for disambiguation of sentence-final words in sentences presented in multi-talker babble noise. The group of high-proficiency HSs was not statistically different from native speakers in their use of contextual information.

While the project was not designed as a correlational study, the outcomes of the SPIN test can be compared with the results of two tests of phonological control and a test of lexical knowledge completed by the same groups of participants. These *post hoc* comparisons are justified given the previous findings regarding the role of L2 phonology and lexical-semantic knowledge in SPIN processing. At the same time, since no direct statistical comparisons were made across the test results, the outcomes are interpreted within the context of what is known about the factors contributing to L2 deficits in SPIN. Reduced L2 phonological sensitivity has an established record of being associated with non-native problems with SPIN processing in non-word sequences (Garcia Lecumberri and Cooke, 2006; Cutler et al., 2008; Broersma and Scharenborg, 2010) and words (Oyama, 1982; Meador et al., 2000; Cooke et al., 2008). The role of lexical-semantic knowledge and a semantic context advantage for native, but not non-native speakers processing SPIN has also been demonstrated (Golestani et al., 2009, 2013; Hervais-Adelman et al., 2014). A comparison of the results of the SPIN test with two tests of phonological control, a low-level phonemic AXB discrimination task and a picture-word discrimination task showed a clear advantage of HSs over L2 learners in all three tasks. Both AXB and the picture-word discrimination task, which tested the robustness of phonolexical representations of minimal pairs of words, targeted the same phonological contrast, the hard-soft consonant distinction that is both difficult for American learners of Russian and very pervasive in Russian speech. Therefore, the performance on this contrast can be considered as a measure of L2 phonological control. Conversely, a multiple choice test of lexical knowledge did not show a heritage advantage over L2 learners. Importantly, in all three tasks, SPIN and two tests of phonological sensitivity, only high-proficiency HSs approximated native performance. The study asks the question whether the SPIN advantage can be explained away by the phonological advantage in HSs. It concludes that phonological sensitivity contributes to the heritage advantage in SPIN, but that more than just phonological sensitivity underlies the heritage advantage. This conclusion is based on the observation that HSs outperform L2 learners on all three tasks in both proficiency ranges, but only high-proficiency HSs are able to use the contextual information to disambiguate the sentence-final word in noise. The study argues that phonological sensitivity and high-level processing involving sentence integration and prediction generation act in a bootstrapping fashion. This leads to a qualitatively different nativelike use of sentence context in noise in the high-proficiency heritage group.

However, at this stage, this conclusion remains tentative, and it invites further research. A promising direction is to continue the work of Aydelott and Bates (2004) by exploring both facilitation and inhibition in non-native processing of speech in adverse conditions, and manipulating the type of masking, low-level energetic versus high-level informational. This will ultimately make it possible to arrive at a better-informed conclusion about the role of top–down processes of sentence integration and generation of predictions about the upcoming word in early and late learners. Another direction in the behavioral and neurolinguistic study of non-native populations, including HSs, is the use of retroactive auditory semantic priming experiments, when the prime word is presented in noise, and the target is either semantically related or unrelated to the prime, e.g., ‘parrot – bird’ is a related pair, and ‘parrot – cake’ is an unrelated one (Golestani et al., 2009; Hervais-Adelman et al., 2014). The participant hears the prime presented in noise, and the target in clear conditions, and must decide which of the two visually presented words was the prime. The facilitative role of the retroactive semantic context in native, but not non-native language was observed in reverse semantic priming experiments for native French speakers listening to French and English word pairs (Golestani et al., 2009). Additionally, only a native language context effect was found in an fMRI study using the same retroactive semantic priming technique (Hervais-Adelman et al., 2014). The use of retroactive semantic priming in noise makes it possible to tease apart prediction from integration of context information and to test non-native use of both in degraded speech recognition. Both these directions have a potential to lead to new insights with regard to heritage and late L2 speech processing.

## Conflict of Interest Statement

The author declares that the research was conducted in the absence of any commercial or financial relationships that could be construed as a potential conflict of interest.

## Footnotes

1 The experiments reported in this publication are part of a larger research project Linguistic Correlates of Proficiency sponsored by the Center for Advanced Study of Language at the University of Maryland (see Long et al., 2012).

2 Here and elsewhere in the text, /ɨ/ refers to the high central (or, more exactly, mixed) unrounded vowel that occurs after hard consonants in Russian. Its phonological status is controversial given that it is in complementary distribution with the vowel /i/ that occurs after soft consonants. The present article follows the position of the Leningrad/Saint Petersburg Phonological School (see Bondarko, 2005) and treats /ɨ/ as a separate phoneme.

3 The information about the Interagency Language Roundtable proficiency scale and the testing format can be found at: http://www.govtilr.org/skills/ILRscale1.htm

4 The diacritic /’/ as in /t’/is conventionally used to mark phonological softness in Russian consonants.

5 The results of the AXB experiment were partially reported in Lukyanchenko and Gor (2011).

6 Mauchly’s test indicated that the assumption of sphericity had been violated (χ = 25.7, *p* < 0.01), therefore the degrees of freedom were corrected using Huynh–Feldt estimates of sphericity (ε = 0.99).

7 The results from the control condition are not reported here.

8 One participant was excluded from the data analysis because they did not follow the task instructions; accordingly, the data analysis was done on 67 subjects.

9 http://corpus.leeds.ac.uk/ruscorpora.html

10 http://www.ruscorpora.ru/en/index.html
